# *MiR-34b* Regulates Muscle Growth and Development by Targeting *SYISL*

**DOI:** 10.3390/cells14050379

**Published:** 2025-03-05

**Authors:** Yuting Wu, Xiao Liu, Yonghui Fan, Hao Zuo, Xiaoyu Niu, Bo Zuo, Zaiyan Xu

**Affiliations:** 1 Key Laboratory of Swine Genetics and Breeding of the Ministry of Agriculture and Rural Afairs, College of Animal Science and Technology, Huazhong Agricultural University, Wuhan 430070, China; wuyuting@webmail.hzau.edu.cn (Y.W.); huangjingqi@webmail.hzau.edu.cn (X.L.); xuwenqi@webmail.hzau.edu.cn (Y.F.); zuohao@webmail.hzau.edu.cn (H.Z.); niuniua@webmail.hzau.edu.cn (X.N.); 2 Key Laboratory of Agriculture Animal Genetics, Breeding and Reproduction of the Ministry of Education, Huazhong Agricultural University, Wuhan 430070, China; 3 Department of Basic Veterinary Medicine, College of Veterinary Medicine, Huazhong Agricultural University, Wuhan 430070, China; 4 Hubei Hongshan Laboratory, Wuhan 430068, China

**Keywords:** *miR-34b*, *SYISL*, myoblast, proliferation, differentiation

## Abstract

Non-coding genes, such as microRNA and lncRNA, which have been widely studied, play an important role in the regulatory network of skeletal muscle development. However, the functions and mechanisms of most non-coding RNAs in skeletal muscle regulatory networks are unclear. This study investigated the function and mechanism of *miR-34b* in muscle growth and development. *MiR-34b* overexpression and interference tests were performed in C2C12 myoblasts and animal models. It was demonstrated that *miR-34b* significantly promoted mouse muscle growth and development in vivo, while *miR-34b* inhibited myoblast proliferation and promoted myoblast differentiation in vitro. Bioinformatics prediction using TargetScan for miRNA target identification and Bibiserv2 for potential miRNA–gene interaction analysis revealed a *miR-34b* binding site in the *SYlSL* sequence. The molecular mechanism of *miR-34b* regulating muscle growth and development was studied by co-transfection experiment, luciferase reporter gene detection, RNA immunoprecipitation, and RNA pull-down. *MiR-34b* can directly bind to *SYISL* and AGO2 proteins and regulate the expression of *SYISL* target genes *p21* and *MyoG* by targeting *SYISL*, thereby regulating muscle growth and development. This study highlights that, as a novel regulator of myogenesis, *miR-34b* regulates muscle growth and development by targeting *SYISL*.

## 1. Introduction

Skeletal muscle represents the most abundant tissue in mammals, constituting roughly 40–50% of total body weight [1]. Skeletal myogenesis is a process in which skeletal muscle myoblast eventually forms multi-core muscle cells and mature muscle fibers after cell proliferation, differentiation, and fusion [2]. The skeletal muscle proliferation and differentiation processes are regulated by a complex network formed by a range of biomolecules [3]. In the stage of myoblast proliferation, the muscle cell cycle is harmoniously regulated by the family of Cyclin and Cyclin-dependent kinases (CDKs) [4]. As a member of the Cip and Kip families, p21 inhibits mammalian cell cycle by inhibiting CDKs activity [5]. In the stage of myoblast differentiation, myogenic myoblasts are mainly regulated by the myogenic regulatory factors (MRFs) family, including myogenic factor 5 (Myf5), myogenic determinants (MyoD), Myogenin (MyoG) and MRF4 [6]. When myoblasts begin to differentiate, the expression of MyoD gradually increases, and, subsequently, MyoD induces the expression of MyoG [7]. As an essential regulator of myogenic differentiation, MyoG promotes the formation of multinucleated myofibers in myoblasts.

MicroRNAs (miRNAs) play an important role in silencing target genes. MiRNAs are defined as small single-stranded RNA molecules 19–24nt in length, that degrade target genes or inhibit protein translation by targeting mRNA [8]. Multiple miRNAs are involved in muscle growth and development, including miR-128 and miR-24–3p that promote muscle differentiation [9,10], miR-491 and miR-690 that inhibit muscle differentiation [11,12], and miR-199a-5p that balance myoblast proliferation and differentiation [13]. The miRNA-34 family (including miR-34a, *miR-34b*, and *miR-34c*) are processed from two distinct primary transcripts: Pri-*miRNA-34b/c* generates both *miR-34b* and *miR-34c* through a shared precursor, while *miR-34a* originates from its independent Pri-miRNA-34a transcript [14]. The *miR-34* family are recognized tumor suppressors inhibiting tumor cell proliferation, migration, and invasion [15,16,17]. Moreover, *miR-34/449* induces cell cycle arrest by targeting cell cycle-related proteins and *miR-34/449* forces epithelial cells to exit the cell cycle and promotes the differentiation of epithelial cells [18].

Our previous studies showed that *SYISL* is a negative regulator of muscle development among mice, pigs, and humans. Mechanistically, *SYISL* facilitates the recruitment of zeste homolog 2 protein, the catalytic subunit of the polycomb repressive complex 2, to the promoter regions of the cell cycle inhibitor p21 and myogenic differentiation markers MyoG. This interaction drives H3K27me3 deposition, thereby epigenetically repressing the transcription of these target genes [19]. *SYISL* knockout in mice resulted in a marked increase in muscle fiber number and muscle mass [20]. In contrast, the overexpression of the *SYISL* gene reduced the muscle fiber cross-sectional area in pig muscle [19,21]. However, whether the role of *SYISL* in muscle is regulated by miRNAs is unclear. In this study, bioinformatics prediction using TargetScan for miRNA target identification and Bibiserv2 for potential miRNA–gene interaction analysis revealed a *miR-34b* binding site in the *SYlSL* sequence. Then, we found that *miR-34b* regulates mouse myoblast proliferation and differentiation by targeting *SYISL*. Our findings provide a new role of *miR-34b* in regulating myogenesis.

## 2. Materials and Methods

### 2.1. Animals

All the C57BL/6J wild-type (WT) mice used for this study were from the Experimental Animal Center of Huazhong Agricultural University. All the animal experiments were conducted in compliance with good laboratory practice standards, ensuring access to a nutritionally balanced diet and sufficient water for the animals. Animal feeding and testing were based on National Research Council Guide for the Care and Use of Laboratory Animals and approved by the Institutional Animal Care and Use Committee at Huazhong Agricultural University. The approval date is 15 September 2015, and the approval number (ID number) is HZAUMO-2015–038.

### 2.2. Overexpression of MiR-34b in Muscles and Phenotype Measurement

Four-week-old wild-type C57BL/6J male mice were used for injection. The quadriceps (Qu), tibialis anterior (TA), and gastrocnemius (Gas) muscles of the right and left leg were injected with *miR-34b* overexpression (*miR-34b* mimics) and negative control (miR-NC) once a week for 4 weeks, and the volume for continuous injection is 50 μL, 50 μL, 100 μL, and 100 μL, respectively. The mice were euthanized by cervical dislocation, and Gas, TA, and Qu muscles of the WT mice were collected and weighed, separately. Data were normalized to the body weight (mg/g). Muscle paraffin sections underwent antigen retrieval in 0.01 M sodium citrate buffer (pH 6.0) at 70 °C for 30 min, followed by membrane permeabilization with 0.1% Triton X-100. Sections were subsequently blocked using P0100B blocking solution (Beyotime Biotechnology, Shanghai, China) at 37 °C for 2 h, then incubated overnight at 4 °C with rabbit anti-dystrophin primary antibody (Abcam, Cambridge, United Kingdom, ab275391, 1:200 dilution). After PBS washes, the specimens were exposed to CY3-conjugated anti-mouse secondary antibody (Beyotime Biotechnology, Shanghai, China) for fluorescent labeling. The images were visualized with a fluorescence microscope (IX51-A21PH, Olympus, Tokyo, Japan). For each muscle slice sample, 150 complete muscle fibers of different fields of view were randomly selected for statistical analysis. ImageJ software (Version 1.53k) was used to calculate the area of muscle fibers.

### 2.3. Cell Isolation, Culture, and Transfection

Myogenic progenitor cells were obtained from 3-week-old C57BL/6J mice following previously described methods [22]. Briefly, the muscles were dissociated and then digested using 2 mg/mL of type I collagenase (C0130; Sigma-Aldrich, St. Louis, MO, USA). The digestion process was terminated with RPMI 1640 medium enriched with 20% fetal bovine serum (FBS; Gibco, Grand Island, NY, USA). Subsequently, the cells were incubated on collagen-coated plates for culture, maintained at 37 °C and 5% CO_2_, and cultured in RPMI 1640 medium enriched with 20% FBS, which was further supplemented with 4 ng/mL of basic fibroblast growth factor, 1% chicken embryo extract, and 1% penicillin-streptomycin. Additionally, C2C12 myoblasts sourced from the Cell Bank of the Chinese Academy of Sciences were cultivated in an incubator maintained at 37 °C and 5% CO_2_. These proliferating cells were grown in Dulbecco’s Modified Eagle’s Medium (DMEM) fortified with 10% FBS. For myogenic differentiation, cells were transferred to DMEM containing 2% horse serum (HS; Gibco, Grand Island, NY, USA). Induction of differentiation occurred once the cells reached 80–90% confluence. The transfection of siRNA (100 pmol) or plasmid (4 μg) was performed using Lipofectamine 2000 (9 μL) (Invitrogen, Carlsbad, CA, USA) according to the manufacturer’s instructions.

### 2.4. Plasmid Construction

According to GenBank in NCBI, the CDS sequences of the *SYISL* genome were obtained. To construct the *SYISL* overexpression plasmid, the full-length sequences were cloned into the pcDNA3.1 plasmid (Addgene, Watertown, MA, USA). For in vitro assay, the CDS sequence of the *SYISL* gene was amplified into the PGEM-3Z in vitro transcription vector. Primer Premier 5.0 software was applied for sequence amplification and primers were synthesized by Sangon Biotech (Shanghai, China).

### 2.5. Cell Immunofluorescence Staining

Cell immunofluorescence staining was performed according to the previous literature [23]. Antibodies included a primary antibody MyHC (sc-376157; 1:200; Santa Cruz Biotechnology, Dallas, TX, USA), MyoG (sc-12732; 1:200; Santa Cruz Biotechnology, Dallas, TX, USA), and a secondary antibody (A0521; goat anti-mouse CY3; Beyotime Biotechnology, Shanghai, China). The EdU staining kit was purchased from Guangzhou Ruibo Biotechnology Co., Ltd. The specific experimental steps can be referred to the instructions of the kit (C10310). The images were visualized with a fluorescence microscope (IX51-A21PH, Olympus, Tokyo, Japan).

### 2.6. Total RNA Preparation and Quantitative Real-Time PCR (RT-qPCR)

Total RNA was isolated using TRIzol reagent (Invitrogen, Carlsbad, CA, USA) according to the manufacturer’s instructions. Reverse transcription was performed with the RevertAid reverse transcriptase (Thermo Scientific, Waltham, MA, USA). RT-qPCR analysis was performed on Applied Biosystems StepOnePlus Real-Time PCR system. The relative RNA expression levels were calculated using the Ct (2^−ΔΔCt^) method as described previously [24]. The complete primer sequences employed for the RT-qPCR analysis are detailed in the table below (Table 1).

### 2.7. Western Blotting

Muscle tissue and cellular proteins were isolated using radioimmunoprecipitation assay buffer supplemented with 1% phenylmethylsulfonyl fluoride (Beyotime Biotechnology, Shanghai, China), followed by Western blotting as previously described [25]. Antibodies that were used included MyoG (sc-12732; 1:200; Santa Cruz Biotechnology, Dallas, TX, USA), MyHC (sc-376157; 1:1000; Santa Cruz Biotechnology, Dallas, TX, USA), β-actin (sc-4777; 1:1000; Santa Cruz Biotechnology, Dallas, TX, USA), CDK6 (BA1513; 1:300; BOSTER, Wuhan, China), Ki67 (ab16667; 1:1000; Abcam, Cambridge, United Kingdom), p21 (BM4382; 1:200; BOSTER, Wuhan, China), and a secondary antibody (1:3000; Santa Cruz Biotechnology, Dallas, TX, USA). Relative protein expression levels were quantified against β-actin as the endogenous loading control, with the densitometric analysis of immunoreactive bands executed through ImageJ software (Version 1.53k) employing standardized grayscale measurement protocols.

### 2.8. Dual-Luciferase Reporter Assay

*SYISL* with mutated or unmutated *miR-34b* binding sites was cloned into the pmirGLO Dual-Luciferase miRNA Target Expression Vectors (E1330; Promega, Madison, WI, USA), respectively. Those reporter vectors were transfected or cotransfected into C2C12 or HeLa cells using Lipofectamine 2000 and then tested according to a previously published method [26]. Luciferase activity was quantified with a PerkinElmer 2030 Multilabel Reader (PerkinElmer, Waltham, MA, USA).

### 2.9. Biotin-Labeled RNA Pulldown

Biotin-labeled RNA pulldown was performed according to a previously published method [19]. The in vitro transcription of the *SYISL* gene was performed according to the instructions of the OMEGA kit (MicroElute RNA Clean Up Kit; Omega Bio-Tek, Norcross, GA, USA). Namely: mix 1 μg of in vitro transcribed RNA with 1 mg of total cell lysate at room temperature, incubate for 1 h by rotation, and then add 30 μL of beads for 1 h by rotation. With the *SYISL* sense strand as the experimental group and the antisense strand as the control group, *miR-34b* was pulled down, respectively. *miR-34b* was used for the RT-qRCR.

### 2.10. RNA Binding Protein Immunoprecipitation (RIP) Assay

RNA immunoprecipitation (RIP) assays were performed with the Magna RIP Kit (Millipore, Burlington, MA, USA) according to the manufacturer’s protocol, utilizing an AGO2-specific antibody (ab3748; Abcam, Cambridge, United Kingdom). Co-precipitated RNAs were analyzed by RT-qPCR, and enrichment was calculated as relative fold change based on a prior methodology [27].

### 2.11. Bioinformatics Pipeline for miRNA Target Prediction

In this study, a multi-step computing pipeline was used in combination with miRBase (http://www.mirbase.org) to perform standardized miRNA sequence verification (*miR-34b*-3p). TargetScan (http://www.targetscan.org) using the context++ score filter (<0.3) was used in conservative seed area prediction, and the Bibiserv2 RNAhybrid module performed the thermodynamic verification of miRNA-mRNA interactions (ΔG < –24 kcal/mol, 3’ complementary pairing).

### 2.12. Statistical Analysis

Experimental measurements were expressed as arithmetic mean ± standard deviation throughout this study, with corresponding sample sizes annotated in respective legends. Statistical analyses between two groups were performed using unpaired or paired Student’s *t*-test. The multiple comparison statistical analysis of data was performed from three or more groups using R software (version 4.1). An initial analysis of variance (ANOVA) was performed using the aov function to assess significant differences between the group means. Specific groups with statistically significant differences were then identified by pairing comparisons using the Tukey HSD feature. For all the analyses, * *p* < 0.05, ** *p* < 0.01, and *** *p* < 0.001 were considered to be statistically significant.

## 3. Results

### 3.1. Effects of miR-34b on Skeletal Muscle Mass and Related Genes

**Figure 1:**
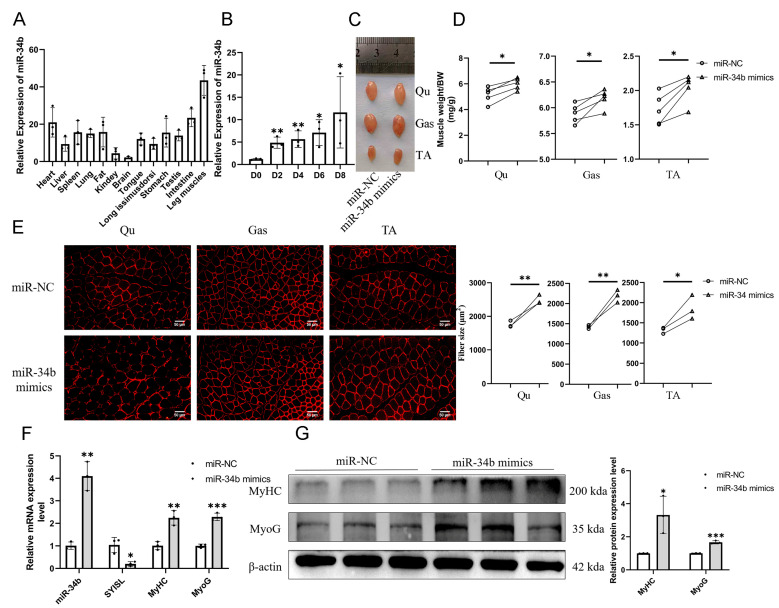
Effects of *miR-34b* on skeletal muscle mass and related genes. (**A**) RT–qPCR showed that *miR-34b* is highly expressed in leg muscle. Data were presented as mean ± SDs, *n* = 3. (**B**) RT-qPCR analysis revealed a progressive upregulation of *miR-34b* expression during C2C12 myoblast differentiation. Data were presented as mean ± SDs, *n* = 3. * *p* < 0.05, ** *p* < 0.01. (**C**) Representative pictures of Qu, Gas, and TA muscles of 2-month-old mice with intramuscular injection of *miR-34b* mimics and miR-NC into the right and left legs of 1-month-old mice. (**D**) Analysis of five independent experiments demonstrated that intramuscular delivery of *miR-34b* mimics significantly enhanced the mass of Qu, Gas, and TA muscles, with data normalized to body weight (mg/g). Data were presented as mean ± SDs, *n* = 5. * *p* < 0.05. (**E**) Immunofluorescence images of dystrophin immunofluorescence staining in Qu, Gas, and TA muscles. Quantitative analysis across three independent experiments revealed that intramuscular *miR-34b* mimics administration significantly increased the mean myofiber cross-sectional area, with ≥150 myofibers analyzed per experiment. Scale bar, 50 μm. Data were presented as mean ± SDs, *n* = 3. * *p* < 0.05, ** *p* < 0.01. (**F**,**G**) Analysis by RT-qPCR (**F**) and Western blotting (**G**) results demonstrated that intramuscular injection of *miR-34b* mimics in mice markedly upregulated the expression levels of *MyHC* and *MyoG*. Data were presented as mean ± SDs, *n* = 3. * *p* < 0.05, ** *p* < 0.01, *** *p* < 0.001.

The expression level of *miR-34b* in 13 different tissues of 2-month-old C57BL/6 mice, including heart, liver, spleen, lung, fat, kidney, brain, tongue, back muscle, stomach, testis, intestine, and leg muscle, was detected by RT-qPCR (Figure 1A). The results showed that the expression of *miR-34b* was the highest in the leg muscle of mice, and it was also expressed in other tissues. Then, we studied the expression changes of *miR-34b* at day 0, 2, 4, 6, and 8 of C2C12 cell differentiation. During the differentiation of C2C12 cells, the expression level of *miR-34b* gradually increases (Figure 1B).

To assess *miR-34b’*s role in modulating muscle mass, we injected *miR-34b* mimics and miR-NC intramuscularly into the right and left legs’ Qu, Gas, and TA muscles of 4-week-old WT mice. The intramuscular injection of *miR-34b* significantly increased both the size and mass of the Qu, Gas, and TA muscles (Figure 1C,D). Immunofluorescence showed that the mean cross-sectional areas of individual myofibers of the Qu, Gas, and TA muscles were significantly increased by the intramuscular injection of *miR-34b* (Figure 1E). RT-qPCR and Western blotting showed that the expression of *MyHC* and *MyoG* was significantly increased by the intramuscular injection of *miR-34b* (Figure 1F,G). These results show that *miR-34b* promotes muscle growth.

### 3.2. Effects of miR-34b on Genes Related to Myoblast Proliferation and Differentiation

**Figure 2:**
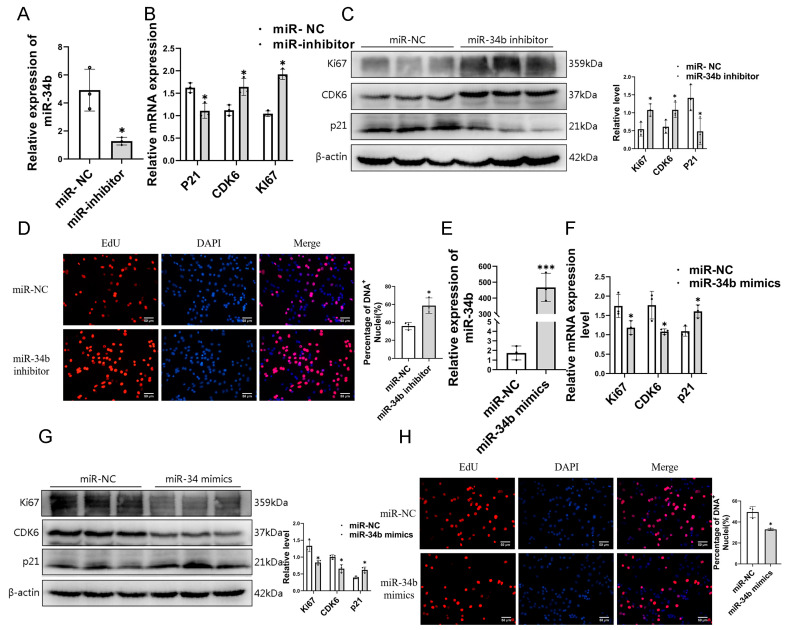
Effects of *miR-34b* on genes related to myoblast proliferation. (**A**) The inhibiting effect of *miR-34b* inhibitor is remarkable. Data were presented as mean ± SDs, *n* = 3. * *p* < 0.05. (**B**) RT-qPCR of proliferated C2C12 myoblasts showed that *CDK6* and *ki67* levels are significantly increased and *p21* level is significantly decreased in *miR-34b* knockdown (*miR-34b* inhibitor) group compared with the negative control (miR-NC) group. Data were presented as mean ± SDs, *n* = 3. * *p* < 0.05. (**C**) Western blotting of proliferated C2C12 myoblasts showed that CDK6 and ki67 protein levels are significantly increased and p21 protein level is significantly decreased in *miR-34b* knockdown (*miR-34b* inhibitor) group compared with the negative control (miR-NC) group. Data were presented as mean ± SDs, *n* = 3. * *p* < 0.05. (**D**) Representative photograph of EdU staining in proliferating C2C12 myoblasts. Quantification of three independent experiments showed that cell proliferation is promoted after inhibiting *miR-34b*. Data were presented as mean ± SDs, *n* = 3 * *p* < 0.05. (**E**) The overexpression effect of *miR-34b* mimics is remarkable. Data were presented as mean ± SDs, *n* = 3. *** *p* < 0.001 (**F**) RT-qPCR of proliferated C2C12 myoblasts shows that *CDK6* and *ki67* levels are significantly decreased and *p21* level is significantly increased in *miR-34b* overexpression (*miR-34b* mimics) group compared with the negative control (miR-NC) group. Data were presented as mean ± SDs, *n* = 3. * *p* < 0.05. (**G**) Western blotting of proliferated C2C12 myoblasts showed that CDK6 and ki67 protein levels are significantly decreased and p21 protein level is significantly increased in *miR-34b* overexpression (*miR-34b* mimics) group compared with the negative control (miR-NC) group. Data were presented as mean ± SDs, *n* = 3. * *p* < 0.05. (**H**) Representative photograph of EdU staining in proliferating C2C12 myoblasts. Quantification of three independent experiments showed that cell proliferation is inhibited after *miR-34b* overexpression. Data were presented as mean ± SDs, *n* = 3. * *p* < 0.05.

We investigated the effect of *miR-34b* on myoblast proliferation. Firstly, we overexpressed and inhibited *miR-34b* with *miR-34b* mimics and inhibitor-transfected C2C12 cells, and the results showed that *miR-34b* was inhibited in C2C12 cells, and total RNA and total protein were extracted after 2 days of culture in proliferation medium (Figure 2A). RT-qPCR and Western blotting were used to detect the expression changes of *CDK6*, *ki67*, and *p21* genes related to myoblast proliferation at mRNA and protein levels, respectively (Figure 2B,C). The results showed that the expression levels of *CDK6* and *ki67* increased significantly, while the expression levels of *p21* decreased significantly. At the same time, the above results were verified by the EdU experiment, which showed that the inhibition of *miR-34b* could promote the proliferation of C2C12 cells (Figure 2D). These results suggest that the inhibition of *miR-34b* promotes the proliferation of C2C12 cells. In order to further verify the effect of *miR-34b* on myoblast proliferation, *miR-34b* was overexpressed in C2C12 cells, and total RNA and total protein were extracted after 2 days of proliferation (Figure 2E). The experimental results showed that the expression levels of *CDK6* and *ki67* decreased significantly, while the expression levels of *p21* increased significantly (Figure 2F,G). These results suggest that the overexpression of *miR-34b* inhibits the proliferation of C2C12 cells. Meanwhile, the EdU results further indicated that *miR-34b* inhibited the proliferation of myoblasts (Figure 2H).

**Figure 3:**
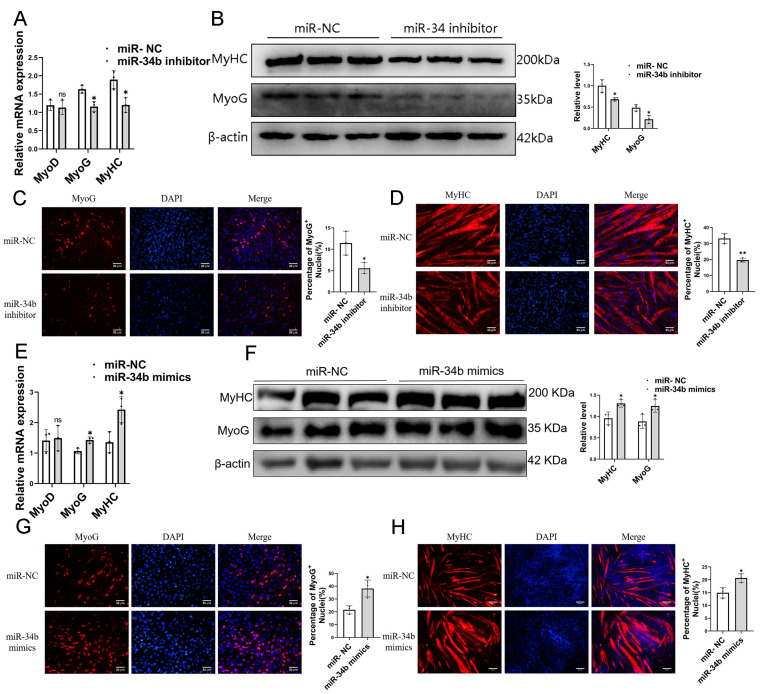
Effects of *miR-34b* on genes related to myoblast differentiation. (**A**) RT-qPCR analysis of differentiated C2C12 myoblasts revealed a marked reduction in *MyoG* and *MyHC* expression levels following *miR-34b* knockdown (*miR-34b* inhibitor) relative to the negative control (miR-NC) group. Data were presented as mean ± SDs, *n* = 3. ns *p* ≥ 0.05, * *p* < 0.05. (**B**) Western blot analysis of differentiated C2C12 myoblasts revealed a marked reduction in MyoG and MyHC protein levels in the *miR-34b* knockdown (*miR-34b* inhibitor) group compared to the negative control (miR-NC). Data were presented as mean ± SDs, *n* = 3. * *p* < 0.05. (**C**,**D**) Representative images of immunofluorescence staining for MyoG (**C**) and MyHC (**D**) in differentiated C2C12 myoblasts and quantification of three independent experiments showed that *miR-34b* knockdown inhibits myoblast differentiation and fusion. Scale bars, 50 μm. Data were presented as mean ± SDs, *n* = 3 * *p* < 0.05, ** *p* < 0.01. (**E**) RT-qPCR of differentiated C2C12 myoblasts showed that *MyoG* and *MyHC* levels are significantly increased in *miR-34b* overexpression (*miR-34b* mimics) group compared with the negative control (miR-NC) group. Data were presented as mean ± SDs, ns *p* ≥ 0.05, *n* = 3. * *p* < 0.05. (**F**) Western blot analysis of differentiated C2C12 myoblasts revealed a marked increase in MyoG and MyHC protein levels in the *miR-34b* overexpression (*miR-34b* mimics) group compared to the negative control (miR-NC). Data were presented as mean ± SDs, *n* = 3. * *p* < 0.05. (**G**,**H**) Representative images of immunofluorescence staining for MyoG (**G**) and MyHC (**H**) in differentiated C2C12 myoblasts and quantification of three independent experiments showed that *miR-34b* overexpression promotes myoblast differentiation and fusion. Scale bars, 50 μm. Data were presented as mean ± SDs, *n* = 3. * *p* < 0.05.

Next, we explored the effect of *miR-34b* on myoblast differentiation. After inhibiting *miR-34b* in C2C12 cells, differentiation was induced for 2 days, and then total RNA and total protein were extracted. RT-qPCR and Western blot were used to detect the expression changes of myoblast differentiation-related genes *MyoG* and *MyHC* at the mRNA and protein levels, respectively (Figure 3A,B). The results showed that after the inhibition of *miR-34b*, the mRNA and protein levels of *MyoG* and *MyHC* decreased, while the mRNA levels of *MyoD* showed no significant change. The above experimental results showed that the inhibition of *miR-34b* could inhibit the differentiation of myoblasts, and then the above results were further verified at the in situ level by immunofluorescence staining (Figure 3C,D). C2C12 cells were induced to differentiate 4 days after the overexpression of *miR-34b*. Immunostaining results showed that the expression levels of MyoG and MyHC decreased after the inhibition of *miR-34b*, which was consistent with the results of the RT-qPCR and Western blot. In order to further verify the effect of *miR-34b* overexpression on myoblast differentiation, C2C12 cells were induced to differentiate for 2 days after the overexpression of *miR-34b*, and then the total RNA and total protein of the cells were extracted (Figure 3E,F). The results showed that after the overexpression of *miR-34b*, at the mRNA level, the mRNA expression levels of *MyoG* and *MyHC* increased, while the expression levels of *MyoD* had no significant change. At the same time, the expression levels of MyoG and MyHC increased significantly (Figure 3G,H). These results suggest that the overexpression of *miR-34b* promotes the differentiation of C2C12 cells. In conclusion, the results showed that *miR-34b* inhibits myoblast proliferation and promotes myoblast differentiation.

### 3.3. MiR-34b Directly Binds to SYISL

**Figure 4:**
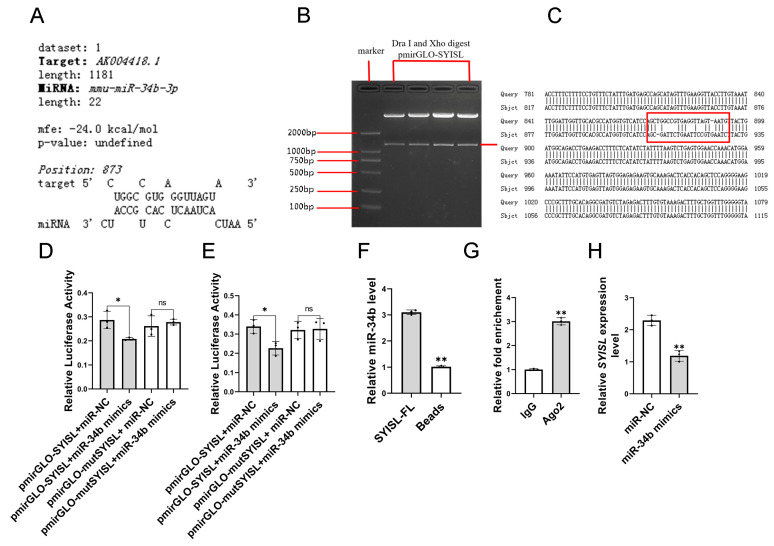
The effect of *miR-34b* on *SYISL*. (**A**) Combined miRNA target prediction analysis using TargetScan and Bibiserv2 bioinformatics tools identified a putative *miR-34b* binding site within the *SYlSL* sequence, with TargetScan providing seed-region complementarity validation and Bibiserv2 confirming thermodynamic stability of the miRNA-mRNA interaction. (**B**) Gel electrophoresis of double digestion of pmirGLO-*SYISL*. (**C**) Non-complementary mutant sequence of *miR-34b* binding site on *SYISL*. (**D**) Dual-luciferase reporter assays showed that *miR-34b* can reduce the dual-luciferase activities of *SYISL* in C2C12. Data were presented as mean ± SDs, *n* = 3. ns *p* ≥ 0.05, * *p* < 0.05. (**E**) Dual-luciferase reporter assays showed that *miR-34b* can reduce the dual luciferase activities of *SYISL* in Hela. Data were presented as mean ± SDs, ns *p* ≥ 0.05, *n* = 3. * *p* < 0.05. (**F**) RNA pull-down experiment showed that *SYISL* binds to *miR-34b*. Data were presented as mean ± SDs, *n* = 3. ** *p* < 0.01. (**G**) RIP experiment showed that *SYISL* is combined AGO2. Data were presented as mean ± SDs, *n* = 3. ** *p* < 0.01. (**H**) RT-qPCR of differentiated C2C12 myoblasts showed that *SYISL* level is significantly decreased in *miR-34b* overexpression (*miR-34b* mimics) group compared with the negative control (miR-NC) group. Data were presented as mean ± SDs, *n* = 3. ** *p* < 0.01.

According to our previous results, *SYISL* is more distributed in the cytoplasm of myoblasts, and *SYISL* promotes the proliferation of myoblasts by inhibiting the expression of *p21*, while its effect on the differentiation of myoblasts is realized by inhibiting *MyoG*. Bioinformatics prediction using TargetScan for miRNA target identification and Bibiserv2 for potential miRNA–gene interaction analysis revealed a *miR-34b* binding site in the *SYlSL* sequence. (Figure 4A). In addition, *miR-34b* and *SYISL* had opposite biological functions, so it was speculated that *miR-34b* acted by targeting *SYISL*. To further verify the binding of *miR-34b* to *SYISL*, we cloned the full-length sequence of *SYISL* into the pmirGLO dual-luciferase reporter vector (Figure 4B). Site-specific mutation primers were designed to carry out site-specific mutation on the site where *SYISL* binds to *miR-34b*, that is, mut*SYISL* was cloned into the pmirGLO dual-luciferase reporter vector (Figure 4C).

We seeded C2C12 cells into 24-well plates and cultured them for 12 h. When the cell plate density reached about 60%, the co-transfection experiment was carried out. The experimental groups were as follows: pmirGLO-*SYISL* + *miR-34b* mimics, pmirGLO-*SYISL* + miR-NC, pmirGLO-mut*SYISL* + *miR-34b* mimics, and pmirGLO-mut*SYISL* + miR-NC. Three biological replicates were set up in each treatment group, and after transfection, proliferation medium was added to culture for 24 h, and then lysed cells were recovered and fluorescence values were measured. The results showed that *miR-34b* could significantly reduce the fluorescence ratio of wild-type *SYISL* double fluorescent vectors. However, when the binding site of *SYISL* was mutated, the fluorescence ratio of wild-type *SYISL* double fluorescent vectors decreased significantly. Compared with miR-NC, the fluorescence value did not change (Figure 4D). These results indicate that this site is indeed the direct binding site between *SYISL* and *miR-34b*. In order to exclude the influence of species and verify the binding of *miR-34b* to *SYISL* in non-mouse cells, human cervical cancer cells (Hela) were selected for the experiment, and the same experimental treatment group and control treatment group were set up for the co-transfection experiment with C2C12 cells (Figure 4E). Finally, the ratio of double fluorescence detected was consistent with the results in C2C12 cells.

In vitro experiments were conducted to verify whether *miR-34b* directly binds to *SYISL*. We seeded C2C12 cells with a 10 cm cell culture dish, then transfected *miR-34b* mimics, labeled the sense strand and antisense strand of *SYISL* with biotin, respectively, and then performed RNA pull-down experiments in C2C12 cells overexpressing *miR-34b*. With the *SYISL* sense strand as the experimental group and the antisense strand as the control group, *miR-34b* was pulled down, respectively, and then the amount of *miR-34b* pulled down was quantitatively detected. The results showed that the amount of *miR-34b* pulled down by the *SYISL* sense strand was significantly higher than that of the *SYISL* antisense strand (Figure 4F). The above experimental results show that *miR-34b* can indeed directly bind to *SYISL*, and at the same time, *miR-34b* can inhibit the expression level of *SYISL*, while miRNA mainly plays the function of RNA silencing through AGO protein. Therefore, the RIP experiment verifies that *SYISL* is combined with AGO2. (Figure 4G). In addition, *miR-34b* mimics were transfected into C2C12 cells, and the expression level of *SYISL* was detected at the proliferation stage and differentiation stage, respectively. The RT-qPCR results showed that the expression level of *SYISL* was significantly decreased after overexpression of *miR-34b* (Figure 4H). In conclusion, the above results prove that *miR-34b* can directly bind to *SYISL* and AGO2 proteins, and *miR-34b* inhibits *SYISL* expression.

### 3.4. MiR-34b Regulates Myoblast Proliferation and Differentiation by Targeting SYISL

**Figure 5:**
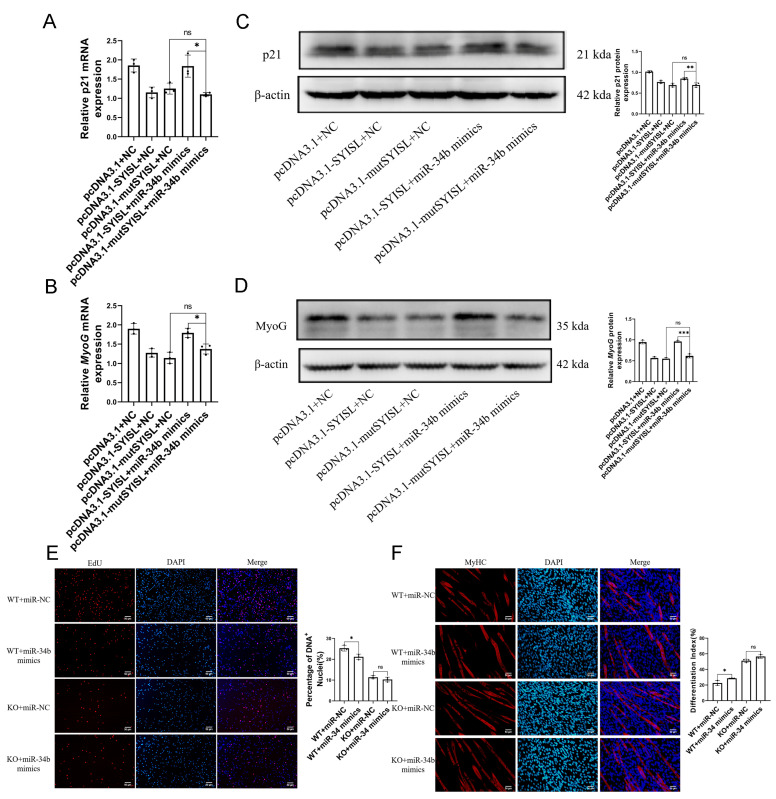
*MiR-34b* regulates myoblast proliferation and differentiation by targeting *SYISL*. (**A**) RT-qPCR of proliferated C2C12 myoblasts showed that *p21* level is significantly decreased in the *SYISL* overexpression vector of *miR-34b* binding site mutation and *miR-34b* mimics co-transmutation treatment group (pcDNA3.1-mut*SYISL* + *miR-34b* mimics) compared with the wild-type *SYISL* overexpression vector and *miR-34b* mimics co-transmutation treatment group (pcDNA3.1-*SYISL* + *miR-34b* mimics). Data were presented as mean ± SDs, *n* = 3. ns *p* ≥ 0.05, * *p* < 0.05. (**B**) RT-qPCR of differentiated C2C12 myoblasts showed that *MyoG* level is significantly decreased in the *SYISL* overexpression vector of *miR-34b* binding site mutation and *miR-34b* mimics co-transmutation treatment group (pcDNA3.1-mut*SYISL* + *miR-34b* mimics) compared with the wild-type *SYISL* overexpression vector and *miR-34b* mimics co-transmutation treatment group (pcDNA3.1-*SYISL* + *miR-34b* mimics). Data were presented as mean ± SDs, *n* = 3. ns *p* ≥ 0.05, * *p* < 0.05. (**C**) Western blotting of proliferated C2C12 myoblasts showed that p21 protein level is decreased in the *SYISL* overexpression vector of *miR-34b* binding site mutation and *miR-34b* mimics co-transmutation treatment group (pcDNA3.1-mut*SYISL* + *miR-34b* mimics) compared with the wild-type *SYISL* overexpression vector and *miR-34b* mimics co-transmutation treatment group (pcDNA3.1-*SYISL* + *miR-34b* mimics). Data were presented as mean ± SDs, *n* = 3. ns *p* ≥ 0.05, ** *p* < 0.01. (**D**) Western blotting of differentiated C2C12 myoblasts shows that MyoG protein level is decreased in the *SYISL* overexpression vector of *miR-34b* binding site mutation and *miR-34b* mimics co-transmutation treatment group (pcDNA3.1-mut*SYISL* + *miR-34b* mimics) compared with the wild-type *SYISL* overexpression vector and *miR-34b* mimics co-transmutation treatment group (pcDNA3.1-*SYISL* + *miR-34b* mimics). Data were presented as mean ± SDs, ns *p* ≥ 0.05, *n* = 3. *** *p* < 0.001. (**E**) Representative images of EdU staining of the proliferation in mouse myogenic progenitor and quantification of three independent experiments showed that cell proliferation was inhibited after *miR-34b* overexpression (WT + *miR-34b* mimics) in wild-type cells compared to the negative control (WT + miR-NC). After *SYISL* knockout, there was no significant difference in cell proliferation after *miR-34b* overexpression (KO + *miR-34b* mimics) compared with negative control (KO + miR-NC). Scale bars, 50 μm. Data were presented as mean ± SDs, ns *p* ≥ 0.05, *n* = 3. * *p* < 0.05. (**F**) Representative images of immunofluorescence staining in differentiated mouse myogenic progenitor and MyHC quantification of three independent experiments showed that overexpression of *miR-34b* (WT + *miR-34b* mimics) promoted cell differentiation compared with negative control (WT + miR-NC). After *SYISL* knockout, there was no significant difference in cell differentiation after *miR-34b* overexpression (KO + *miR-34b* mimics) compared with negative control (KO+miR-NC). Scale bars, 50 μm. Data were presented as mean ± SDs, *n* = 3. ns *p* ≥ 0.05, * *p* < 0.05.

We proved that *miR-34b* inhibited cyclin by promoting the expression of *p21*, thus inhibiting myoblast proliferation. The regulation of differentiation is to promote myoblast differentiation by promoting the expression of *MyoG*. At the same time, further experiments proved that *miR-34b* could directly bind to *SYISL* and inhibit the expression of *SYISL*. Therefore, we speculated that *miR-34b* exerts its regulatory function through targeting *SYISL*. We used the *SYISL* overexpression vector and *SYISL* overexpression vector with the mutant *miR-34b* binding site in C2C12 cells to perform co-transfection experiments with miR-NC or *miR-34b* mimics. At the proliferation stage of C2C12 cells, the target gene of *SYISL*, *p21*, was detected by RT-qPCR. The results showed that, compared with the wild-type *SYISL* overexpression vector and *miR-34b* mimics co-transmutation treatment group, when the binding site of mutant *SYISL* and *miR-34b* was detected, overexpressed *miR-34b* cannot bind to mutant *SYISL*, so it cannot restore the *SYISL* inhibition of *p21* by targeting *SYISL* (Figure 5A). At the same time, the *SYISL* target gene *MyoG* was detected at the differentiation stage of C2C12 cells, and the results showed that compared with the wild-type *SYISL* overexpression vector and *miR-34b* mimics co-transmutation group, when the binding site of mutated *SYISL* and *miR-34b* was detected, overexpressed *miR-34b* also could not bind to mutant *SYISL*, and thus could not restore *SYISL’*s inhibition of *MyoG* by targeting *SYISL* (Figure 5B). At the same time, the protein level was detected, and the results showed that the mRNA and protein levels were consistent (Figure 5C,D). The EdU staining of mouse myogenic progenitor proliferation showed that after *SYISL* knockout, the inhibitory effect of *miR-34b* overexpression on cell proliferation was removed (Figure 5E). Representative images of immunofluorescence staining and the quantification of MyHC in differentiated mouse myogenic progenitor showed that after *SYISL* knockout, the role of *miR-34b* in promoting cell differentiation after overexpression was eliminated. These results suggest that *miR-34b* regulates the expression of *SYISL* target genes *p21* and *MyoG* by targeting *SYISL*, thereby regulating the proliferation and differentiation of myoblasts. In conclusion, we found that *miR-34b* can regulate myoblast proliferation and differentiation by inhibiting *SYISL* (Figure 5F).

## 4. Discussion

Non-coding RNAs have crucial roles in the growth and development of skeletal muscle [28,29]. In our previous study, we identified a novel long non-coding RNA-*SYISL*, which is distributed in cytoplasm and nuclei. In addition, we found that *SYISL* functions as a sponge of miR-103-3p and miR-205-5p to promote muscle atrophy [21]. In this study, we confirmed *miR-34b* can directly bind to *SYISL* and regulate *SYISL* expression. At present, *miR-34* is generally regarded as a tumor suppressor miRNA, which plays a synergistic role with tumor suppressor p53 [30]. In addition, miR-34 family members also play a role in regulating the cell cycle. After knocking out *miR-34a*, *miR-34b*, and *miR-34c* in mice, the expression of Ccnd1, Ccnb1, CDK6, and other cell cycle proteins increased significantly in epithelial cells, and multiple epithelial cells proliferated. Normal mouse respiratory epithelial cells differentiate into cilia on embryonic day 16.5, while *miR-34* knockout mouse respiratory epithelial cells remain proliferative and unable to exit the cell cycle [18]. In this study, we also demonstrated the dual function of *miR-34b* to inhibit myoblast proliferation and promote myoblast differentiation.

At present, the most reported miRNAs exert regulatory functions by targeting mRNA, inhibiting mRNA abundance and mRNA translation [31,32]. It is also commonly reported that lncRNAs, acting as “molecular sponges” for miRNAs, bind miRNAs by competing with mRNAs targeted by miRNAs. However, miRNAs also exert regulatory functions by targeting lncRNAs. miR-103 can significantly reduce the expression level of *lncWDR59* and inhibit the proliferation of vascular endothelial cells by targeting *lncWDR59*. After designing antisense oligonucleotides for miR-103 and *lncWDR59* binding sites, miR-103 cannot reduce the expression of *lncWDR59* and inhibit endothelial cells proliferation [33]. The targeted inhibition of miR-103 to *lncWDR59* can be considered as an RNA silencing effect. MiRNA derived from incomplete complementary double-stranded stem ring precursor cut by Dicer enzyme binds to AGO protein, recognizes target RNA through base pairing, and performs an RNA silencing function [34]. In this study, we verified the direct combination of *miR-34b* and *SYISL* through bioinformatics prediction, a dual-luciferase reporter vector experiment, and an RNA pull-down experiment. By the intramuscular injection of *miR-34b* mimics into the leg muscles of mice, we found that muscle growth was significantly promoted. By the cotransfection of the *miR-34b* mimics *SYISL* overexpression vector or mutant *SYISL* hyperexpression vector and *miR-34b* mimics in C2C12 cells, we found that *miR-34b* had no effect on the expression levels of *p21* and *MyoG* when *SYISL* lost the *miR-34b* binding site. And by transfection *miR-34b* mimics in wild-type and *SYISL* knockout muscle progenitors, respectively, we found that *miR-34b* had no effect on myogenic progenitor cell proliferation and differentiation when *SYISL* was knocked out. These results confirmed our conjectures that *miR-34b* targets *SYISL* directly and regulates muscle growth and development.

In animal husbandry, *miR-34b* promotes muscle growth and development, thereby promoting meat yield and quality, providing a new direction for the development of animal husbandry. In terms of clinical application, the incidence of human diseases such as sarcopenia continues to increase with age. *miR-34b* provides a new way to treat muscle diseases by targeting the negative muscle regulatory factor *SYISL*.

## 5. Conclusions

In this study, we identified *miR-34b* that has a significant effect on muscle growth and development. *MiR-34b* plays an important role in the proliferation and differentiation of myoblasts. Mechanistically, *miR-34b* directly interacts with *SYISL*, weakening its regulation of myoblast proliferation and differentiation. Our study identified *miR-34b* as a novel regulator of myogenesis and provided its molecular regulatory mechanism by targeting *SYISL*.

## Figures and Tables

**Table 1:** All primers used in RT-qPCR.

Primer	Sequence	Tm (°C)
*SYISL*	F: CTCGTGGTCCCTCCCTGTAA	60
	R: GTCTGCGTGCTCCTGTGGTT	
*MyHC*	F: CAAGTCATCGGTGTTTGTGG	60
	R: TGTCGTACTTGGGCGGGTTC	
*MyoG*	F: CCATCCAGTACATTGAGCGCCTACA	60
	R: ACGATGGACGTAAGGGAGTGCAGAT	
*MyoD*	F: CGAGCACTACAGTTGGCGACTAAGA	60
	R: GCTCCACTATGCTGGACAGGCAGT	
*β-actin*	F: GCCTCACTGTCCACCTTCCA	60
	R: AGCCATGCCAATGTTGTCTCTT	
*CDK6*	F: GGCGTACCCACAGAAACCATA	60
	R: AGGTAAGGGCCATCTGAAAACT	
*Ki67*	F: ATCATTGACCGCTCCTTTAGGT	60
	R: GCTCGCCTTGATGGTTCCT	
*p21*	F: ATGTCCAATCCTGGTGATGTCC	60
	R: AGTCAAAGTTCCACCGTTCTCG	
*miR-34b*	F: GCGCGAATCACTAACTCCACT	60
	R: AGTGCAGGGTCCGAGGTATT	
*U6*	F: GCTTCGGCAGCACATATACT	60
	R: TTCACGAATTTGCGTGTCAT	

All primers used in RT-qPCR are shown in the table.

## Data Availability

The original contributions presented in the study are included in the article/Supplementary Materials, and further inquiries can be directed to the corresponding author.

## Supplementary Materials

The following supporting information can be downloaded at https://www.mdpi.com/article/10.3390/cells14050379/s1: Supplementary Table S1. Sequence of miRNA oligonucleotides. Supplementary Table S2. qPCR program.

## Abbreviations

The following abbreviations are used in this manuscript:

miRNA	microRNA
Myf5	myogenic factor 5
MyoD	myogenic determinants
MyoG	Myogenin
MyHC	Myosin Heavy Chain
CDKs	Cyclin-dependent kinases
WT	wild-type
Qu	quadriceps
Gas	gastrocnemius
TA	tibialis anterior
DMEM	Dulbecco’s Modified Eagle’s Medium
HS	horse serum
RIP	RNA binding protein immunoprecipitation
FBS	fetal bovine serum
SD	standard deviation
RT-qPCR	quantitative real-time PCR
BW	body weight
Hela	human cervical cancer cells
