# Supplementary material for: MiR-34b Regulates Muscle Growth and Development by Targeting SYISL
Source: Cells. 2025 Mar 5;14(5):379. doi: 10.3390/cells14050379 (PMC11898696; doi:10.3390/cells14050379)
Supplement: Supplementary file 1 [file cells-14-00379-s001.zip › cells-3469632-supplementary.pdf]

Supplementary Materials:

Table S1. Sequence of miRNA oligonucleotides.

miRNA	Sequence(5′-3′)
mmu-miR-34b-3p	AATCACTAACTCCACTGCCATC
miR-34b mimics	AAUCACUAACUCCACUGCCAUC
	UGGCAGUGGAGUUAGUGAUUUU
miR-34b NC	UUCUCCGAACGUGUCACGUT
	TACGUGACACGUUGGAGAATT
miR-34b inhibitor	GAUGGCAGUGGAGUUAGUGAUU
miR-34b inhibitor NC	CAGUACUUUUGUGUAGUACAA

Table S2. qPCR program

Step	Temperature(°C)	Time(s)	Number of cycle
1	95	300	1
2	95	10	40
3	60	30	40
4	72	30	40
